# Irradiation Decreases the Neuroendocrine Biomarker Pro-Opiomelanocortin in Small Cell Lung Cancer Cells *In Vitro* and *In Vivo*

**DOI:** 10.1371/journal.pone.0148404

**Published:** 2016-02-05

**Authors:** Suzanne L. Meredith, Jennifer L. Bryant, Muhammad Babur, Philip W. Riddell, Roya Behrouzi, Kaye J. Williams, Anne White

**Affiliations:** 1 Faculty of Medical and Human Sciences, University of Manchester, Manchester, M13 9PT, United Kingdom; 2 Faculty of Life Sciences, University of Manchester, Manchester, M13 9PT, United Kingdom; Texas Tech University Health Sciences Center, UNITED STATES

## Abstract

**Background:**

Small cell lung cancer (SCLC) is an extremely aggressive disease, commonly displaying therapy-resistant relapse. We have previously identified neuroendocrine and epithelial phenotypes in SCLC tumours and the neuroendocrine marker, pro-opiomelanocortin (POMC), correlated with worse overall survival in patients. However, the effect of treatment on these phenotypes is not understood. The current study aimed to determine the effect of repeated irradiation treatment on SCLC cell phenotype, focussing on the neuroendocrine marker, POMC.

**Results:**

Human SCLC cells (DMS 79) were established as subcutaneous xenograft tumours in CBA nude mice and then exposed to repeated 2Gy irradiation. In untreated animals, POMC in the blood closely mirrored tumour growth; an ideal characteristic for a circulating biomarker. Following repeated localised irradiation *in vivo*, circulating POMC decreased (p< 0.01), in parallel with a decrease in tumour size, but remained low even when the tumours re-established. The excised tumours displayed reduced and distinctly heterogeneous expression of POMC compared to untreated tumours. There was no difference in the epithelial marker, cytokeratin. However, there were significantly more N-cadherin positive cells in the irradiated tumours. To investigate the tumour response to irradiation, DMS79 cells were repeatedly irradiated *in vitro* and the surviving cells selected. POMC expression was reduced, while mesenchymal markers N-cadherin, β1-integrin, fibroblast-specific protein 1, β-catenin and Zeb1 expression were amplified in the more irradiation-primed cells. There were no consistent changes in epithelial marker expression. Cell morphology changed dramatically with repeatedly irradiated cells displaying a more elongated shape, suggesting a switch to a more mesenchymal phenotype.

**Conclusions:**

In summary, POMC biomarker expression and secretion were reduced in SCLC tumours which regrew after irradiation and in repeatedly irradiation (irradiation-primed) cells. Therefore, POMC was no longer predictive of tumour burden. This highlights the importance of fully evaluating biomarkers during and after therapy to assess clinical utility. Furthermore, the gain in mesenchymal characteristics in irradiated cells could be indicative of a more invasive phenotype.

## Introduction

Lung cancer is the leading cause of cancer death in the Western world and small cell lung cancer (SCLC) is the most aggressive form, accounting for around 15% of all cases [1]. This poor prognosis is due to rapid growth, early development of distant metastases and almost inevitable relapse with therapy-resistant disease [2]. The current standard treatment for SCLC is a combination of chemotherapy and radiotherapy. Once the primary tumours and metastases become unresponsive to treatment, survival time for patients is extremely short. The lack of efficacy of chemotherapy and radiotherapy after SCLC relapse highlights the importance of gaining a more in-depth understanding of the cellular and molecular changes in tumours deemed therapy resistant.

Currently, radiotherapy is administered to SCLC patients who present with limited disease and often also in extensive disease. Radiotherapy is given in the first or second cycle of chemotherapy in either once or twice daily doses for 3–5 weeks [3]. Radio- and chemo-resistance can be mimicked *in vitro* and studies have shown that irradiation-resistant SCLC cells also acquire resistance to other agents [4,5]. However, the phenotypic characteristics of irradiation-resistant SCLC cells have not been documented. Neuroendocrine markers have proven useful, but are often limited in their detection and staging of SCLC patients; therefore, more sensitive and reliable biomarkers are sought to strengthen diagnosis and prognosis.

Excess circulating POMC, the precursor of the stress hormone, adrenocorticotrophic hormone (ACTH), is most commonly documented in patients with pituitary tumours but also in patients with non-pituitary tumours, particularly SCLC [6–9]. These patients can present with mild to moderate symptoms of Cushing’s Syndrome. We have shown that circulating levels of the neuroendocrine marker, POMC, correlate with a lower survival rate in patients with SCLC tumours [10]. However, whether this biomarker would be as useful in predicting tumour relapse after treatment is not known.

Non-SCLC (NSCLC) cells treated with radiotherapy or chemotherapy have the capacity to undergo epithelial to mesenchymal transition (EMT) in response to the therapy [11–17] and this is becoming a more widely recognised characteristic of metastasis in many cancer types [18,19]. Less is known about whether there is a similar progression in SCLC or whether EMT is linked to therapy-resistance in this cancer. However, studies have shown that there are subpopulations of adherent SCLC cells *in vitro* that are more mesenchymal and exhibit increased chemoresistance [20]. In addition, there is evidence of EMT in SCLC tumours, which has been linked to increased invasiveness and chemoresistence [21].

The nature of phenotypic transitions and the role of the neuroendocrine phenotype in SCLC tumours are poorly understood. In addition, the impact of irradiation treatment on tumour phenotype has not been described in SCLC. Our aim was to determine whether POMC could act as a biomarker of tumour burden after irradiation treatment or if it is altered as a consequence of irradiation resistance, using the same murine model as previously established [10]. We found that POMC was significantly reduced in resistant cells *in vitro* and *in vivo* and was not a good predictor of tumour regrowth after irradiation. There was also a distinct mesenchymal switch *in vitro* and *in vivo* after irradiation which could be indicative of more aggressive or motile tumour cells.

## Methods

### Cell culture

DMS 79 is an SCLC cell line originally from a pleural effusion taken from a 65 year old male Caucasian and established *in vitro* by Dr Pettengill, (Dartmouth Medical School, Hanover, NH, USA). The cell line was donated by her in 1990 [22]. Cells were authenticated by the DNA Sequencing Facility (University of Manchester) at the time of the study and possess both *p53* and *Rb1* mutations. DMS 79 cells grow as loosely suspended aggregates and were cultured in RTISS media (RPMI 1640 + L-Glutamine supplemented with 2.5% FBS, 5μg/ml insulin, 10μg/ml transferrin, 30nM sodium selenite and 1% HEPES buffer)[23].

DMS 79 cells were irradiated using a fractional x-ray irradiation machine (Faxitron X-ray corporation, Switzerland) at 2Gy and left to recover for 14–21 days. This procedure was repeated for 10 cycles with viability assessments conducted before and 72 hours after each treatment. Total time in culture was 32 weeks and total number of passages of cells was between 30 and 40. Cell viability and doubling times were assessed by CellTiterGlo Luminescent Cell Viability Assay (Promega, WI, USA). Cells were exposed to 5Gy IR challenges to determine whether the cells had gained resistance to the higher doses of irradiation. Irradiation cycles were conducted on 3 individual cultures for each experiment.

### Xenograft studies

#### Ethics Statement

All procedures involving animals were performed in accordance with the UK Home Office Animal (Scientific Procedures) Act, 1986, and approved by the local University of Manchester Ethical Review Committee.

DMS 79 cells were injected subcutaneously into CBA nude female mice at 5x10^6^ cells/mouse in 0.1ml of serum free RPMI-1640 medium with the addition of 50% matrigel. Mice were sourced from the University of Manchester Biological Services Facility and housed in internal ventilation cages in groups of 5, at 22°C, under sterile conditions with autoclaved sawdust bedding and environmental enrichment. The mean weight was 24.5g +/- 0.37g and mice were 12 weeks old at the start of the study. Animals were subjected to 12:12 light dark cycle and *ad libitum* access to sterile standard chow diet (SDS) and sterile water. Mice were allocated treatment groups of 5 animals/group based on time taken for the tumour to reach 250mm^3^. Tumours were locally irradiated or untreated at 250mm^3^ at 2Gy/day for 3, 5 or 10 consecutive days, giving total doses of 6, 10 and 20Gy. All animals were closely monitored for general health and weight daily. All animals remained in good health throughout the experiment unless stated. During the *in vivo* study one mouse was culled mid-experiment due to a swollen abdomen and significant weight loss (from group 4 [10 consecutive IR days]) and was therefore excluded from analysis.

Mice were sacrificed at 10:00am by rising CO_2_ and cervical dislocation when tumours reached 1000mm^3^. Tumours and other organs of interest were then half snap frozen and half formalin fixed and paraffin wax embedded. Blood samples of maximum 80μl were taken by tail nick at days 0, 13 and every 7^th^ day thereafter. Mouse plasma was analysed for POMC by ELISA (see below).

### POMC ELISA

Circulating POMC levels were measured in mouse plasma using a specific two-site ELISA. This assay followed the same protocol as described previously [10]. The lower limit of assay sensitivity during the study was 15pmol/L.

### Immunohistochemistry/Immunocytochemistry

5μm sections of formalin fixed wax embedded DMS 79 tumours were stained for POMC (N1C11, antibody produced in our lab) (see [10] for further details of antibody), neuron-specific enolase (NSE) (mouse monoclonal, clone BBS/NC/VI-H14, Dako cat number M0873, dilution 1:100) cytokeratin (mouse monoclonal, clone AE1/AE3, Dako, cat number M3515, dilution 1:100) and N-cadherin (CDH2 mouse monoclonal, clone 6G11, Dako, cat number M3613, dilution 1:50) using diaminobenzene (DAB) chromagen envision system (Dako). Secondary antibody used was a polyclonal goat anti-mouse IgG conjugated to HRP (Dako, Cat number P0447, dilution 1:200) Antigen retrieval was performed on all sections using citrate buffer (pH6) at 95˚C for 30 minutes.

### Image analysis

Tumour sections were scanned at 20x magnification using an Aperio CT scanner (Aperio Systems, Vista, CA) and cytoplasmic DAB staining was quantified using the Aperio Positive Pixel Count program v9.1 (Aperio Systems, Vista, CA). Only viable tissue from each section was analysed and areas of folded/damaged tissue and necrosis/pre-necrosis were excluded to reduce bias. Negative controls were conducted for all treatment groups in order to determine background staining levels which could then be excluded using the Aperio software. The viable cell areas from whole tumour sections were analysed, tumours from all mice were included in the analysis and 3 sections from each tumour were analysed.

Data generated was expressed as a percentage of the number of positive pixels compared to total number of pixels analysed (positive number fraction), which can be equated to the positive area fraction [24].

### Quantitative PCR

DMS 79 cells exposed to a total of 21Gy IR *in vitro* (8x2Gy cycles and 1x5Gy cycle) showing distinct morphological changes from untreated cells cultured over the same period were harvested. RNA was extracted using a QIAGEN RNeasy Mini Kit (using Qiashredder tubes). cDNA synthesis was carried out using the QuantiTect Reverse Transcription Kit (Qiagen). Primers were obtained from Eurofins MWG, (London, UK) and were designed across exon boundaries. Genes of interest include; *POMC*, *ENO2* (neuron specific enolase [NSE]), *NCAM1* (neural cell-adhesion molecule [N-CAM]), *CHGA* (Chromogranin A [CgA]), *KRT18* (cytokeratin 18 [CK18]), *KRT19* (cytokeratin 19 [CK19]) *CDH1* (epithelial cadherin [E-Cad]), *EPCAM* (Epithelial cell adhesion molecule), *CDH2* (neural cadherin [N-Cad]), *ITGB1* (Integrin β1), *CTNNB1* (β-Catenin), *ZEB1*, *FSP1* (fibroblast-specific protein 1) and *ACTA1* (α-smooth muscle actin). Quantitative PCR was conducted using FastStart Universal SYBR Green (Roche) and run/analysed using StepOnePlus Real Time PCR machine and software (Applied Biosystems).

### Statistical & Data Analysis

All statistical data analysis was carried out using GraphPad Prism software version 5. Data presented is mean and standard error of the mean of a minimum of three individual experiments. Correlation coefficients were assessed using Spearmans correlation test. Quantitative real time PCR data was analysed using StepOne software and Microsoft Excel. Gene expression statistical significance evaluation was measured using unpaired T tests. One-way ANOVA statistical tests were used to compare multiple groups of data including Bonferroni’s multiple comparisons.

## Results

### POMC is a less effective biomarker of tumour growth after irradiation

**Fig 1:**
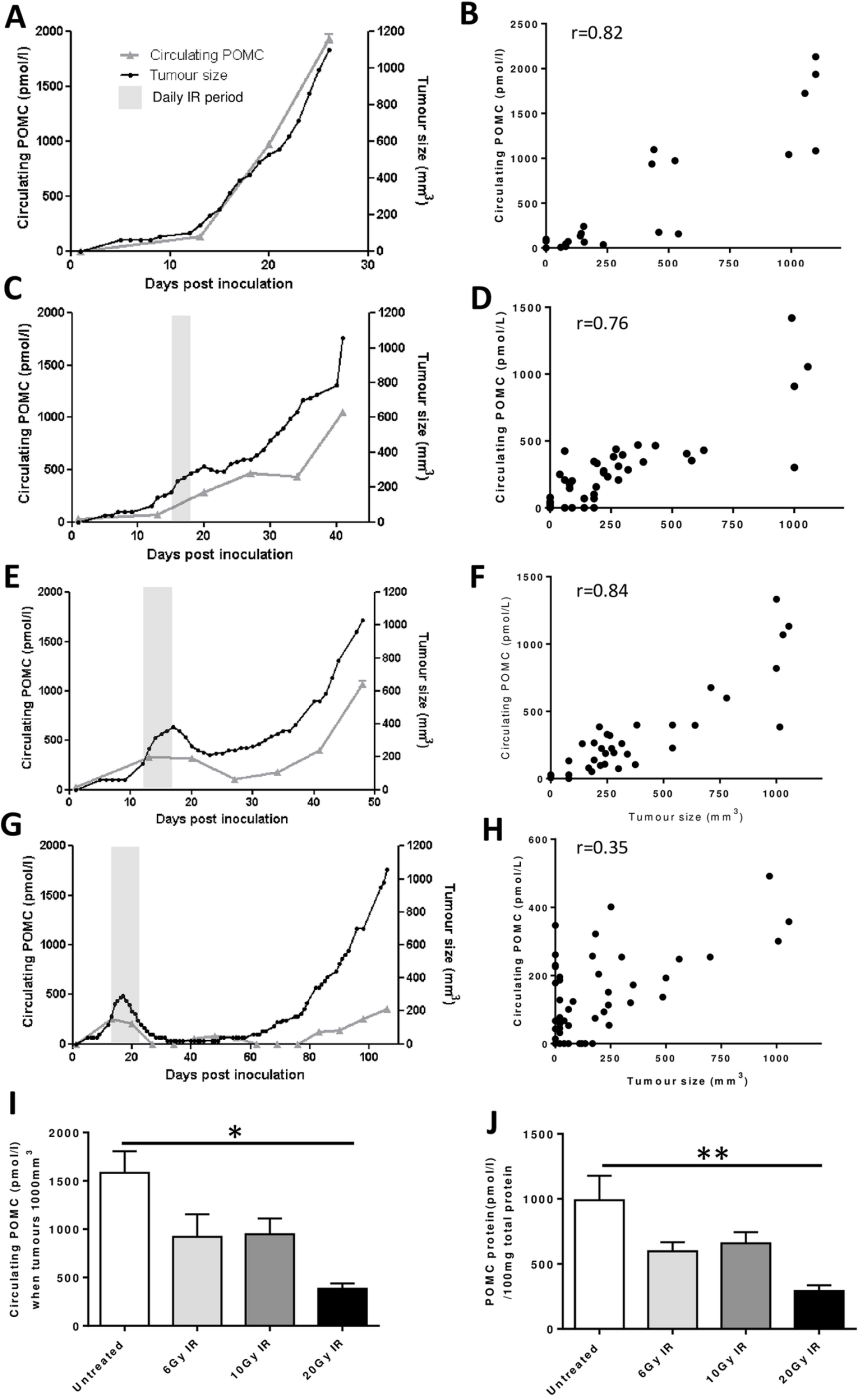
Growth of SCLC xenografts and circulating POMC in response to localised irradiation. DMS 79 cells were established as subcutaneous xenografts and once they were 200–250mm^3^ either left to grow untreated (A) or exposed to 2Gy IR/day for 3 consecutive days (C), 5 consecutive days (E) or 10 consecutive days (G). Circulating POMC was monitored by blood sampling on days 0, 13, 20 and every 7 days thereafter. Shaded bars indicate the period where tumours were locally exposed to IR. Circulating POMC and tumour size were analysed by correlation analysis, which was conducted on all time points from each group (B, D, F, H). Results presented (A, C, E, G) are individual mice but represent 3–5 mice/group. All individual animal data are presented in S1 Fig Circulating POMC was analysed in terminal samples (I). Whole protein from tumour samples was analysed by ELISA for POMC (J). The following numbers of animals reached 1000mm^3^ tumour volume within 120 days from tumour implant; Untreated group 5/5, 3x2Gy 4/5, 5x2Gy 5/5, 10x2Gy 3/4 *p = <0.05 **p = <0.01. Controls in Fig 1A have originally been described in Stovold et al. British Journal of Cancer [10].

Circulating POMC accurately mimicked tumour progression in untreated xenografted mice (Fig 1A) and there was a strong correlation (r = 0.82, Fig 1B). When tumours were locally irradiated for 3 and 5 days there was also a strong positive correlation between tumour size and circulating POMC (Fig 1C–1F). However terminal POMC concentrations were lower than those in untreated animals even though all animals had been maintained until the tumours reached the same size. Even more striking, when subcutaneous DMS 79 tumours were irradiated for 10 consecutive days at 2Gy/day, POMC did not mirror tumour re-growth (r = 0.35, Fig 1G and 1H). Circulating POMC from terminal plasma samples, when tumours were all at 1000mm^3^, was 4 fold lower in the 20Gy irradiation group compared to those in the untreated group (p = 0.0165 Fig 1I). POMC tumour protein was also lower in the 20Gy irradiated group (p = 0.0092 Fig 1J). Data from all individual mice are shown in S1 Fig.

### POMC is decreased and distinctly heterogeneous after repeated irradiation *in vivo*

**Fig 2:**
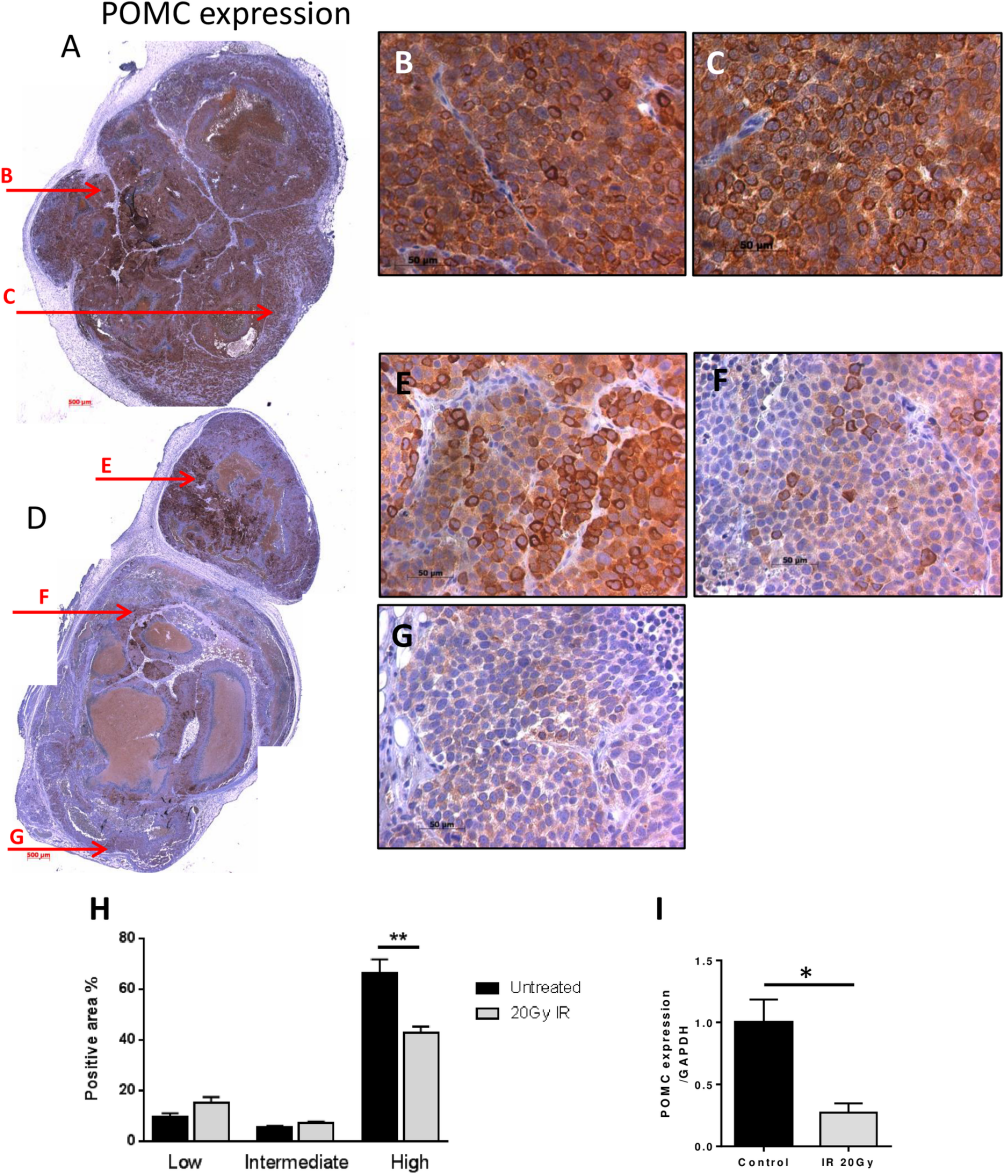
POMC heterogeneity in xenograft tumours after irradiation. DMS 79 cells were established as subcutaneous tumours in nude mice and either left untreated (A-C) or exposed to IR for 10 consecutive days at 2Gy/day (D-G). A central section of the tumour was stained for POMC using our own N1C11 antibody. (A&D) show entire tumour cross-sections, (B&C) show strong homogeneous POMC staining in separate tumour areas in untreated DMS 79 xenografts. (E-G) show viable tumour cell regions of an irradiated DMS 79 xenograft revealing (E) strong staining, (F) weaker heterogeneous staining, (G) weak staining. (H) Quantitive assessment by positive pixel analysis of untreated tumours and 20Gy IR treated tumours stained for POMC. (I) POMC gene expression was analysed in all untreated and 20Gy IR tumours. Tumours presented are representative of 3–5/group with all other tumours in S2 Fig.

Untreated tumours stained uniformly positive for POMC (Fig 2A–2C). However, tumours exposed to 2Gy/day for ten days displayed less and distinctly heterogeneous POMC staining within viable cell regions (Fig 2D–2G). Sections from all tumours are shown in S2 Fig Positive pixel analysis across all tumours confirmed that there were 23% more strongly positive POMC cells in the untreated tumours than in the 20Gy irradiated tumours (Fig 2H). POMC gene expression was also decreased in the 20Gy IR tumours (Fig 2I). When examining an alternative neuroendocrine marker, neuron specific enolase (NSE), there was no difference in staining between the untreated and irradiated tumours (S3 Fig).

### Increased N-Cadherin *in vivo* after irradiation

**Fig 3:**
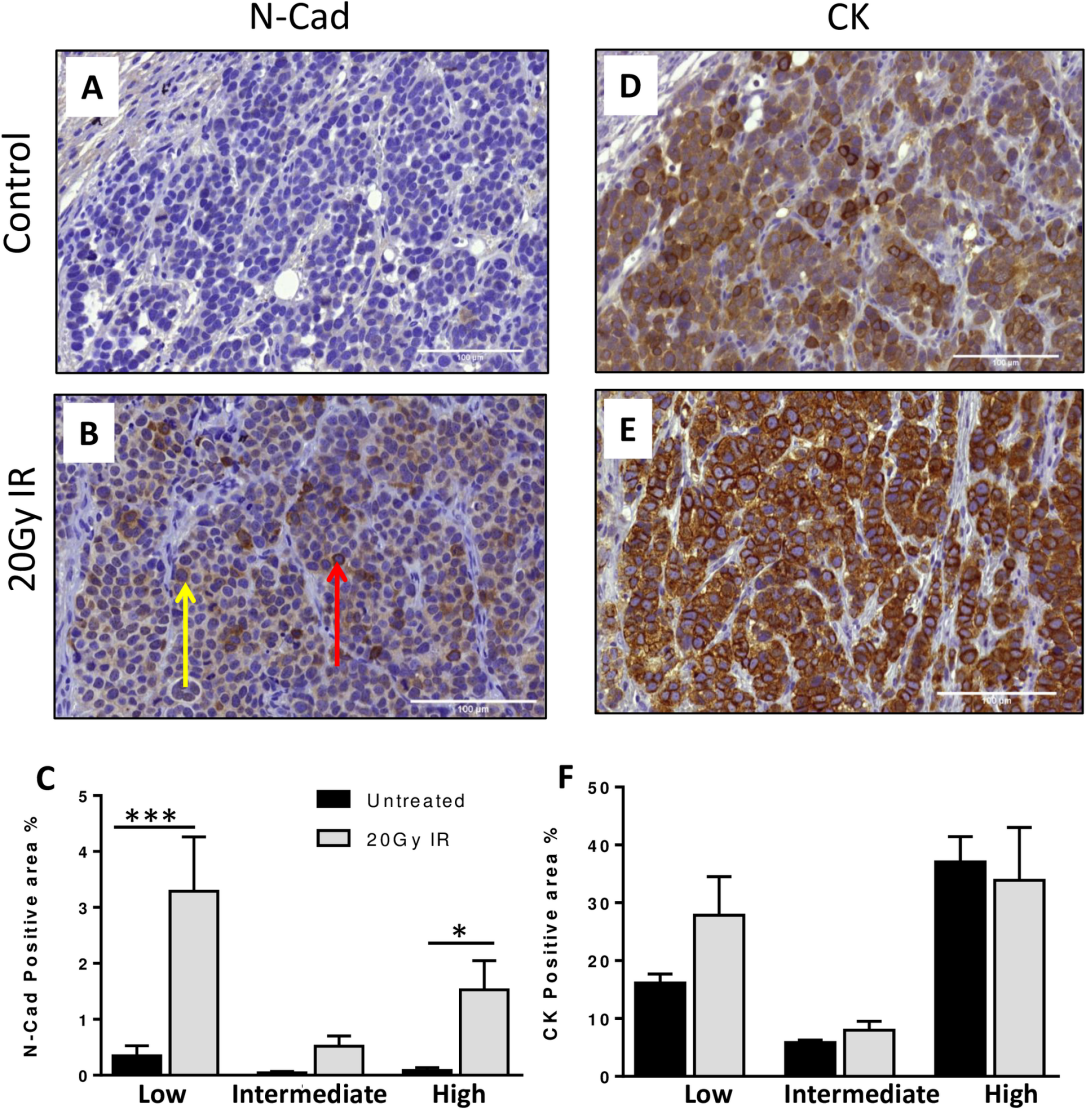
Increased N-Cadherin staining in irradiated tumours. The mesenchymal marker N-Cadherin (N-Cad) (A&B) and the epithelial marker cytokeratin (CK) (D&E) were analysed in all untreated and all 20Gy irradiated tumour sections. Quantitative assessment ([C] N-Cad, [F] CK) by Positive Pixel analysis of stained tissue sections. Staining was quantified as low, intermediate or high in the viable tumour area of untreated (black bars) vs irradiated tumours (grey bars). Red arrow indicates an example of a highly stained cell, yellow arrow indicates an area of low staining. Very low level background staining was discounted. ***p = <0.001 *p = <0.05.

To determine whether there was any additional change in phenotype, irradiated DMS 79 xenograft tumours were also analysed for N-cadherin and cytokeratin (Fig 3). N-cadherin appeared low or absent in untreated tumours but expression was stronger in repeatedly irradiated tumours, with the presence of individual strongly positive N-cadherin cells distributed across the tumour sections (Fig 3A and 3B). In addition, some areas were more densely populated with positive cells, creating a heterogeneous distribution overall. Positive pixel analysis confirmed N-cadherin was significantly increased in irradiated tumours (low staining 9 fold increase p = <0.001, high staining 18 fold increase p = 0.0268) when compared to untreated tumour sections (Fig 3C). Cytokeratin staining, in contrast, showed uniformly positive staining in untreated and irradiated xenograft sections (Fig 3D and 3E), confirmed by positive pixel analysis (Fig 3F).

### No changes in POMC in DMS79 cells after a single dose of irradiation

**Fig 4:**
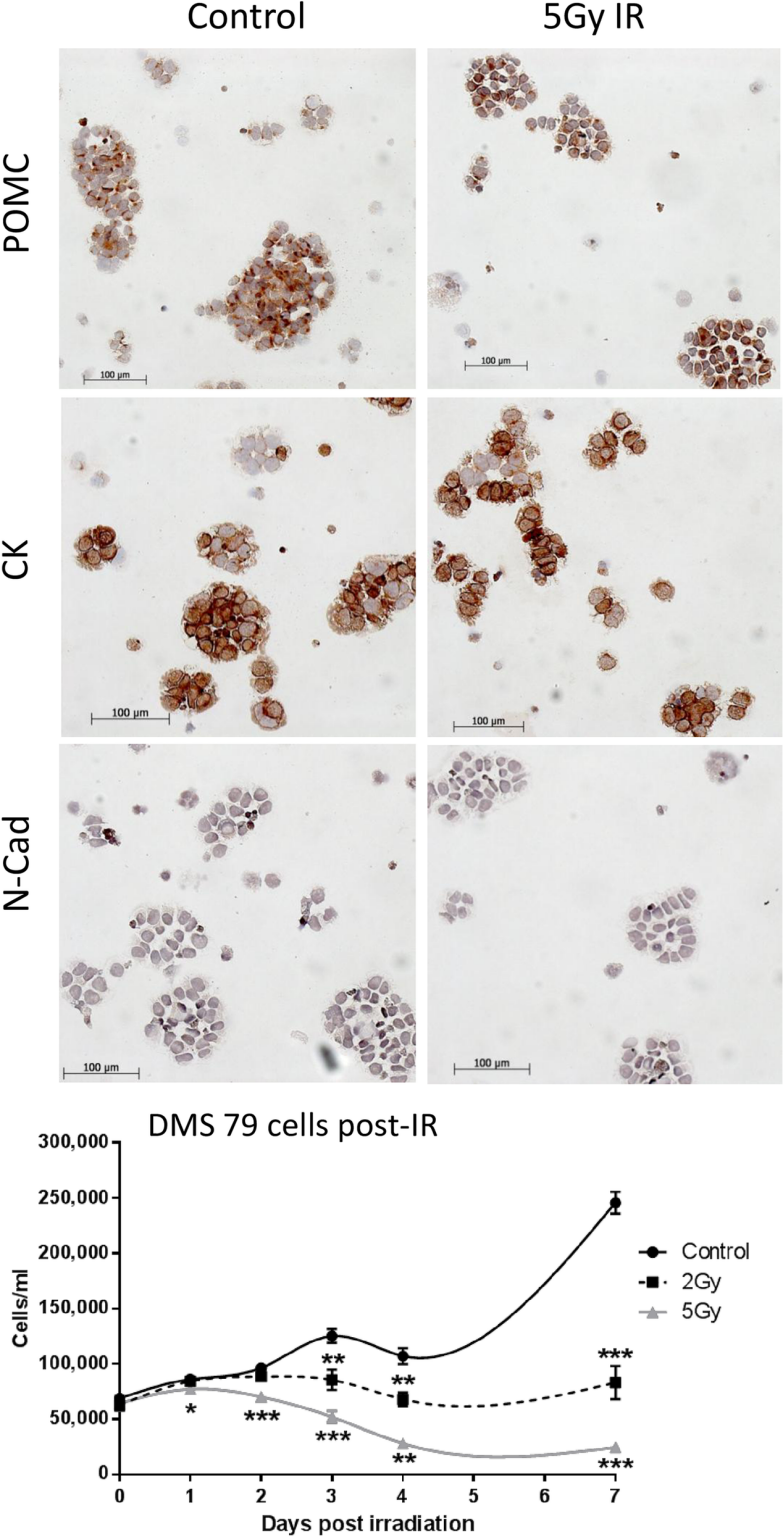
DMS 79 phenotype is not altered after one irradiation cycle in vitro. DMS 79 cells were counted on day 0 and then irradiated at 2Gy and 5Gy. Viable counts were conducted up to 7 days post-irradiation before cells were stained for POMC, cytokeratin (CK) and N-Cadherin. All irradiation cycles were conducted on 3 independent cultures. * p = <0.05 ** p = <0.01 *** p = <0.001

Results from the *in vivo* studies suggested that tumours post-radiation have acquired phenotypic characteristics that differ from untreated tumours and that these changes were coupled with a decrease in POMC biomarker secretion and expression and an increase in N-Cadherin. To investigate this further, DMS79 cells *in vitro* were exposed to radiation and changes in phenotype were monitored. Initially DMS 79 cells were given one dose of 2Gy or 5Gy irradiation to determine whether a single dose was enough to promote any phenotypic changes (Fig 4).

Cell viability was significantly reduced in cells that were irradiated at both 2Gy and 5Gy. However, the surviving viable cells displayed no obvious differences in morphology or in expression of POMC, cytokeratin and N-cadherin, 7 days after irradiation, suggesting that repeated irradiation is coupled with the phenotypic change *in vivo*.

### Modifying radiation phenotype *in vitro*

**Fig 5:**
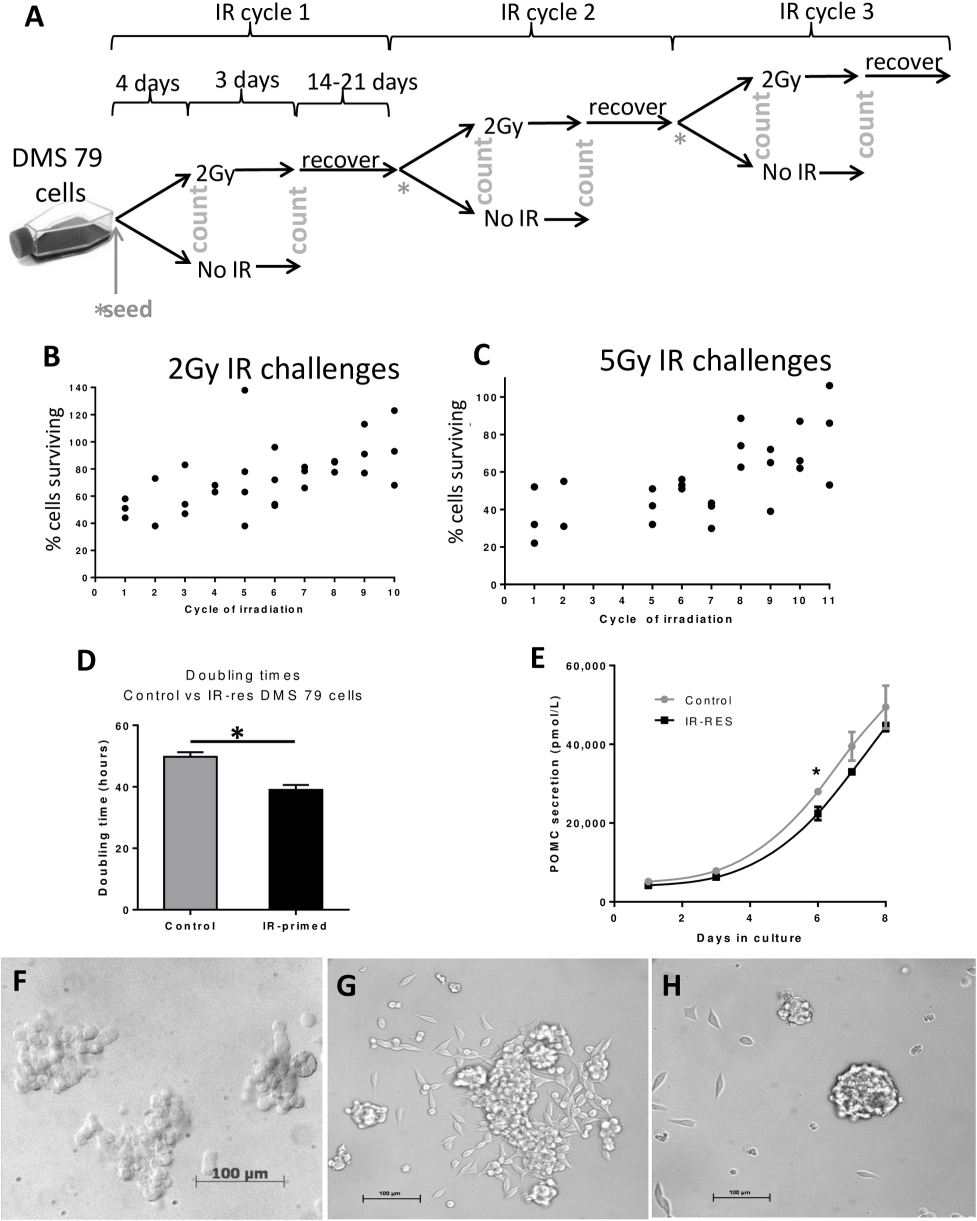
IR-primed DMS 79 cells acquire resistance to irradiation in vitro. DMS 79 cells were irradiated at 2Gy for 10 progressive cycles (schematic [A]). The percentage of cells surviving in the irradiated cultures was compared to ‘control’ ie cells not treated at that particular cycle (B) (Spearman correlation p = <0.0001). The experiment was conducted with these controls to allow for the increased proliferation that irradiated cells displayed over time compared to completely untreated cells. DMS 79 cells were also challenged with higher doses of IR (5Gy) at various points to evaluate radiosensitivity (C). Cells remaining were expressed as a percentage of controls (B&C). Cell doubling times of untreated vs 21Gy IR cells (9 total cycles, 8 of 2Gy and 1 of 5Gy) were calculated over 5 days (D). POMC secretion was assessed over 8 days by POMC ELISA and results were normalized to cell number (E). Cell morphology of untreated DMS 79 cells (F). Cell morphology of cells irradiated to 21Gy, displaying adherent colonies and individual adherent elongated cells (G) and very tightly suspended clusters (H) compared to the loose suspended clumps shown in (F).

DMS 79 cells grown *in vitro* were then subjected to repeated irradiation over 8 months until they became more tolerant to the treatment (Fig 5A schematic). With more cycles of irradiation the number of cells surviving a 2Gy IR challenge increased (Fig 5B). After 9 cycles of irradiation there was a decrease in the doubling time of cells subjected to irradiation (Fig 5D) and a decrease in POMC secretion (Fig 5E).

After each cycle of 2Gy irradiation, some of the cells were taken for challenge with 5Gy to determine whether there was any change in tolerance to greater doses of radiation (Fig 5C). The percentage of surviving cells post-treatment (2Gy and 5Gy) increased as the number of IR cycles increased (Fig 5B and 5C). Cells that had been irradiated to a total of 21Gy (IR-primed cells) showed resistance to further treatment.

Interestingly, IR-primed cells displayed dramatically altered morphology. Untreated DMS 79 cells form irregular suspended aggregates of rounded cells (Fig 5F), whereas IR-primed cultures presented as a combination of adherent clusters, single adherent elongated cells (Fig 5G and 5H) and very tight suspended spheres (Fig 5H). This change in morphology suggests that the cells may have undergone a phenotypic switch, conceivably to a more mesenchymal state.

### IR-primed cells have decreased POMC expression

**Fig 6:**
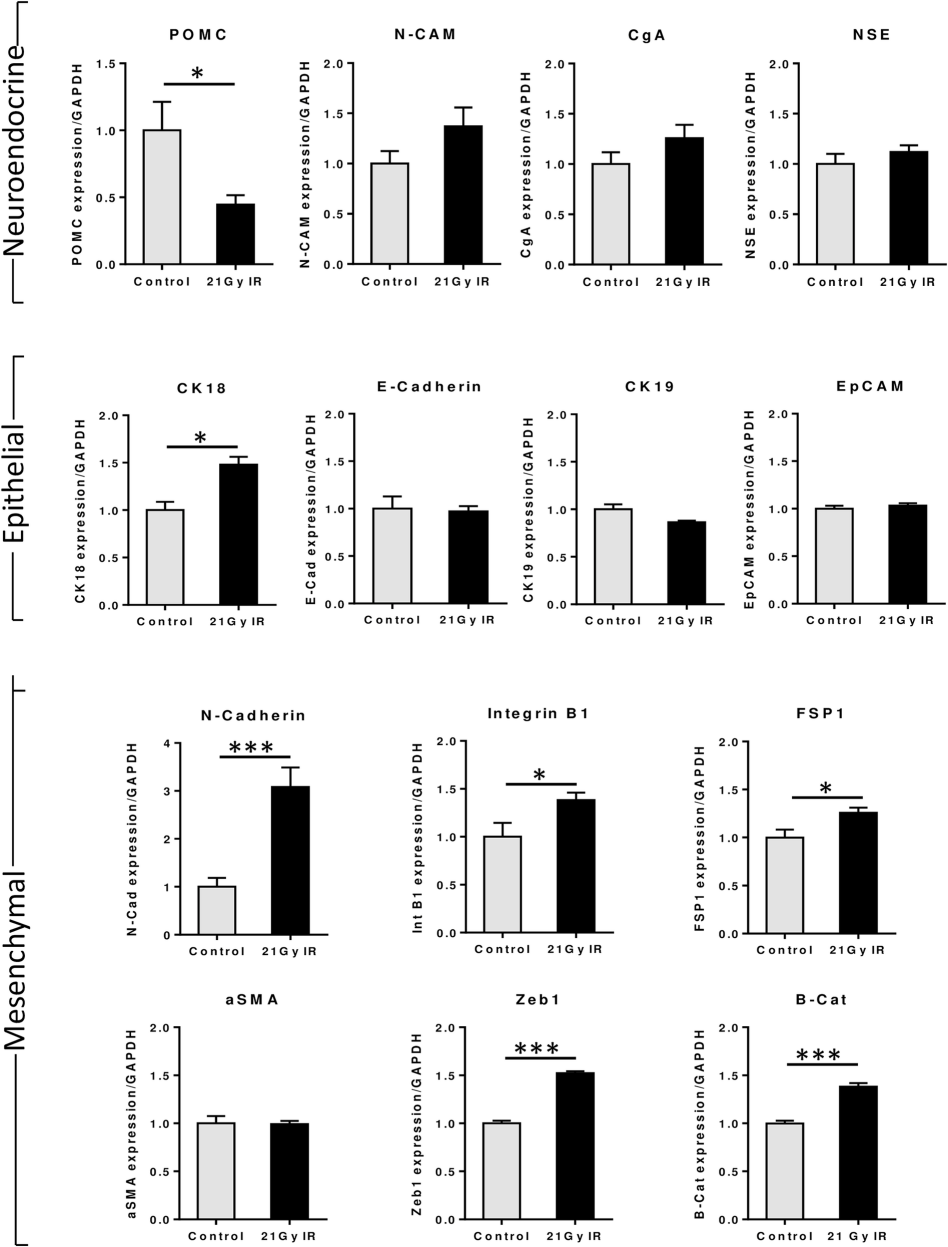
IR primed DMS 79 cells have reduced POMC gene expression. mRNA expression was analysed in DMS 79-IR cells (grey bars) and untreated cells which were grown in parallel conditions to the treated cells (black bars). mRNA levels were normalised to GAPDH expression. *p<0.05 ***p = <0.001.

DMS 79 IR-primed cells that had been subjected to fractionated radiotherapy to a total dose of 21Gy showed a significant decrease in POMC mRNA (p = 0.033). However, there was no change in the other neuroendocrine markers, neural-cell adhesion molecule (N-CAM), chromogranin A (CgA) and neuron-specific enolase (NSE) (Fig 6).

### Radiation treatment is coupled with a switch to a more mesenchymal phenotype

In parallel with the *in vivo* data, the IR-primed SCLC cells showed no consistent overall change in the epithelial markers, cytokeratin 18, cytokeratin 19, E-cadherin and Epithelial cell adhesion molecule (EpCAM) (Fig 6). However, more interestingly, five of the six mesenchymal markers analysed, were upregulated; N-cadherin p = <0.001, Integrin β1 p = 0.044, fibroblast-specific protein 1 p = 0.03, Zeb1 p = <0.001, β-catenin p = <0.001. This data is consistent with the morphological observations *in vitro* and the quantification of *in vivo* staining.

## Discussion

Assessing SCLC tumour cells *in vivo* and *in vitro* has identified a change in phenotype following exposure to repeated irradiation. POMC expression decreased in tumours *in vivo* after repeated irradiation and decreased POMC was also found in irradiated tumour cells *in vitro*. In addition to this altered neuroendocrine phenotype, an increase in mesenchymal characteristics was observed following repeated irradiation.

Numerous studies have identified candidate biomarkers for various cancer types, signifying their importance in diagnosis, prognosis and the early detection of relapse in patients. However, there is significant complexity in the usefulness of biomarkers. In the current study, irradiation of DMS 79 cells *in vivo* resulted in decreased POMC in the blood, and decreased POMC protein and mRNA expression levels in tumours even though the tumours had regrown. This indicates that biomarker expression has changed as a result of treatment and is no longer able to accurately predict tumour regrowth. We have previously described POMC as a novel biomarker in patients with SCLC and showed it correlated with liver metastases [10]. It could be that in the patients, irradiation is reducing POMC expression/production in the primary tumour, (as seen in the xenografts) but any metastases in the patients (especially those in the liver) may still be secreting high levels of POMC into the circulation.

POMC could be a very specific and sensitive biomarker for a subset of SCLC patients who have not been treated. However, it is important to consider that POMC measurement may prove misleading for the majority of patients who have had irradiation and relapsed, because the circulating concentrations of POMC produced by the primary tumour remain suppressed. Therefore POMC shows a markedly differing relationship with tumour volume in untreated versus irradiated tumours. Although it is true that a rise in POMC would indicate relapse following radiotherapy, the tumour burden with which this is associated would be much greater than that observed without radiation treatment. In Fig 1 an untreated tumour of 200 mm3 produces 300pmol/l of POMC but a tumour treated with 20Gy would be 1000cm3 before it produced the same amount of POMC. If this translated to patients, it would influence how well POMC could be used because by the time a relapsed tumour given radiotherapy is big enough to be detected by POMC, it could be beyond the size for which an alternative intervention might be useful. Similar scenarios are likely to exist for other biomarkers, where studies have not considered the type and extent of treatment.

Despite the fact that there was a significant decrease in POMC *in vitro* and *in vivo* after irradiation, there was no change in N-CAM, NSE or chromogranin A gene expression *in vitro*. This indicates that aspects of the neuroendocrine phenotype persist after irradiation. This would suggest that the repeated irradiation may be resulting in changes to the POMC gene or the POMC pathway which lead to decreased POMC secretion, but is not altering the neuroendocrine phenotype in general. These results demonstrate that when analysing a novel biomarker, it is extremely important to be aware of plasticity of biomarker expression post-treatment.

In parallel with the changes in POMC expression there was an upregulation of the mesenchymal markers N-Cadherin, β1-integrin, Zeb1, Fibroblast-specific protein 1 and β-Catenin at the mRNA level. This provides insight into the mechanisms involved in the response of the tumour cells to treatment. Epithelial to mesenchymal transition is known to be associated with resistance to therapy. In this study the acquired resistance is accompanied by a decrease in POMC and an upregulation of mesenchymal markers. Resistance to irradiation of SCLC cells has previously been identified with N-acetylglucoaminyltransferase V (Gnt-V) over-expression and upregulation of Gnt-V *in vivo* causing an increase in N-cadherin, vimentin and ZEB2, again suggesting a link between radiosensitivity and EMT-like change [25]. N-cadherin levels are known to increase after irradiation in NSCLC cells [16,17] and in follicular thyroid xenograft tumours [26]. EMT is also activated in breast cancer cells receiving low-dose radiation [27]. In addition, we observed that the SCLC cells when exposed to repeated rounds of irradiation *in vitro* were more detached from other cells and displayed an elongated morphology which is in keeping with a mesenchymal phenotype.

Although irradiation is significantly reducing tumour burden in patients, it could be driving cells towards a more aggressive, mesenchymal phenotype. The mechanism for this is not known, although reactive oxygen species (ROS) generated as a consequence of radiotherapy or continued smoking can alter cell adhesion and stimulate cell invasion [28]. ROS can also induce EMT through upregulation of the E-cadherin repressor, Snail [29]. However, this mechanism is less likely as E-cadherin levels were unchanged in the irradiated cells in our *in vitro* model. The unchanged E-Cadherin gene expression suggests that rather than a classical EMT mechanism that is often observed in NSCLC [11,12,14,15], the SCLC cells are only upregulating mesenchymal characteristics. It is the mesenchymal phenotype that is considered responsible for the invasion and secondary colonisation of tumour cells [30]. In this particular model, however, irradiation treatment failed to produce metastases in lungs, brains or livers of these mice (data not shown). The lack of metastasis in the *in vivo* model could be attributed to the host environment, difficulties in inter-species signaling (human SCLC cells in mice), lack of immune factors, or insufficient time for cells to colonise secondary organs. Given that there are very few subcutaneous tumour models that spontaneously metastasise, an alternative method of inoculation may be important to develop this further.

Overall, this study has identified an important change in expression of POMC with irradiation both *in vivo* and *in vitro*. This indicates that changes in biomarkers in response to treatment need to be investigated thoroughly to understand their plasticity and the implications for their use. This decrease in a specific neuroendocrine marker is accompanied by a mesenchymal switch in SCLC tumours *in vivo*. Although irradiation is causing tumour cell death in patients, repeated exposure may leave some tumour cells with an altered phenotype and a greater propensity for intravasation and future therapy resistance. Uncovering associations between phenotype switching and therapy-resistance could help determine the best line of treatment for patients with different stages of SCLC.

## Supporting Information

S1 Fig SCLC xenograft growth of all individual mice in response to localised irradiation. Individual mouse data for DMS 79 xenografts. DMS 79 cells were established as subcutaneous xenografts and once they were 200-250mm^3^ either left to grow untreated (A-D) or exposed to 2Gy IR/day for 3 consecutive days (E-H), 5 consecutive days (I-L) or 10 consecutive days (M-O). Circulating POMC was monitored by blood sampling on days 0, 13, 20 and every 7 days thereafter. Shaded bars indicate the period where tumours were locally exposed to IR.
(TIF)

S2 Fig POMC staining in all xenograft tumours. DMS 79 cells were established as subcutaneous tumours in nude mice and either left untreated or exposed to IR for 10 consecutive days at 2Gy/day. A central section of the tumour was stained for POMC using our own N1C11 antibody. In two mice the tumours did not regrow after treatment so POMC could not be assessed (one from group 2 [3 consecutive IR days] and one from group 4 [10 consecutive IR days). Quantitive assessment by positive pixel analysis of untreated tumours and 20Gy IR treated tumours stained for POMC is presented in Fig 2.
(TIF)

S3 Fig Unchanged NSE expression in untreated and irradiated tumours. DMS 79 cells were established subcutaneously in nude mice and either left untreated or exposed to IR for 10 days at 2Gy/day. A central section of the tumour was stained for neuron specific enolase (NSE). Tumours presented are representative of 3-5/group.
(TIF)

## References

[1] Riaz SP, Luchtenborg M, Coupland VH, Spicer J, Peake MD, Moller H (2012) Trends in incidence of small cell lung cancer and all lung cancer. Lung Cancer 75: 280–284. 10.1016/j.lungcan.2011.08.004 21893364

[2] Kalemkerian GP, Akerley W, Bogner P, Borghaei H, Chow L, Downey RJ, et al (2011) Small cell lung cancer. J Natl Compr Canc Netw 9: 1086–1113. 21975911 10.6004/jnccn.2011.0092

[3] Komaki R, Paulus R, Ettinger DS, Videtic GM, Bradley JD, Glisson BS, et al (2012) Phase II study of accelerated high-dose radiotherapy with concurrent chemotherapy for patients with limited small-cell lung cancer: Radiation Therapy Oncology Group protocol 0239. Int J Radiat Oncol Biol Phys 83: e531–536. 10.1016/j.ijrobp.2012.01.075 22560543 PMC3377848

[4] Davey RA, Locke VL, Henness S, Harvie RM, Davey MW (2004) Cellular models of drug- and radiation-resistant small cell lung cancer. Anticancer Res 24: 465–471. 15152945

[5] Henness S, Davey MW, Harvie RM, Davey RA (2002) Fractionated irradiation of H69 small-cell lung cancer cells causes stable radiation and drug resistance with increased MRP1, MRP2, and topoisomerase IIalpha expression. Int J Radiat Oncol Biol Phys 54: 895–902. 12377343 10.1016/s0360-3016(02)03037-7

[6] Gibson S, Ray DW, Crosby SR, Dornan TL, Jennings AM, Bevan JS, et al (1996) Impaired processing of proopiomelanocortin in corticotroph macroadenomas. Journal of Clinical Endocrinology and Metabolism 81: 497–502. 8636257 10.1210/jcem.81.2.8636257

[7] Stewart PM, Gibson S, Crosby SR, Pennt R, Holder R, Ferry D, et al (1994) ACTH precursors characterize the ectopic ACTH syndrome. Clinical Endocrinology 40: 199–204. 8137518 10.1111/j.1365-2265.1994.tb02468.x

[8] Oliver RL, Davis JR, White A (2003) Characterisation of ACTH related peptides in ectopic Cushing's syndrome. Pituitary 6: 119–126. 14971736 10.1023/b:pitu.0000011172.26649.df

[9] Page-Wilson G, Freda PU, Jacobs TP, Khandji AG, Bruce JN, Foo ST, et al (2014) Clinical utility of plasma POMC and AgRP measurements in the differential diagnosis of ACTH-dependent Cushing's syndrome. J Clin Endocrinol Metab 99: E1838–1845. 10.1210/jc.2014-1448 25013995 PMC4184073

[10] Stovold R, Meredith S, Bryant J, Babur M, Williams K, Dean E, et al (2013) Neuroendocrine and epithelial phenotypes in small-cell lung cancer: implications for metastasis and survival in patients. Br J Cancer 108: 1704–1711. 10.1038/bjc.2013.112 23519056 PMC3668479

[11] Kawata M, Koinuma D, Ogami T, Umezawa K, Iwata C, Watabe T, et al (2012) TGF-beta-induced epithelial-mesenchymal transition of A549 lung adenocarcinoma cells is enhanced by pro-inflammatory cytokines derived from RAW 264.7 macrophage cells. J Biochem 151: 205–216. 10.1093/jb/mvr136 22161143

[12] Chen XF, Zhang HJ, Wang HB, Zhu J, Zhou WY, Zhang H, et al (2012) Transforming growth factor-beta1 induces epithelial-to-mesenchymal transition in human lung cancer cells via PI3K/Akt and MEK/Erk1/2 signaling pathways. Mol Biol Rep 39: 3549–3556. 10.1007/s11033-011-1128-0 21713404

[13] Bryant JL, Britson J, Balko JM, Willian M, Timmons R, Frolov A, et al (2012) A microRNA gene expression signature predicts response to erlotinib in epithelial cancer cell lines and targets EMT. Br J Cancer 106: 148–156. 10.1038/bjc.2011.465 22045191 PMC3251842

[14] Pirozzi G, Tirino V, Camerlingo R, Franco R, La Rocca A, Liguori E, et al (2011) Epithelial to mesenchymal transition by TGFbeta-1 induction increases stemness characteristics in primary non small cell lung cancer cell line. PLoS One 6: e21548 10.1371/journal.pone.0021548 21738704 PMC3128060

[15] Shintani Y, Okimura A, Sato K, Nakagiri T, Kadota Y, Inoue M, et al (2011) Epithelial to mesenchymal transition is a determinant of sensitivity to chemoradiotherapy in non-small cell lung cancer. Ann Thorac Surg 92: 1794–1804; discussion 1804. 10.1016/j.athoracsur.2011.07.032 22051275

[16] Gomez-Casal R, Bhattacharya C, Ganesh N, Bailey L, Basse P, Gibson M, et al (2013) Non-small cell lung cancer cells survived ionizing radiation treatment display cancer stem cell and epithelial-mesenchymal transition phenotypes. Mol Cancer 12: 94 10.1186/1476-4598-12-94 23947765 PMC3751356

[17] Jung JW, Hwang SY, Hwang JS, Oh ES, Park S, Han IO (2007) Ionising radiation induces changes associated with epithelial-mesenchymal transdifferentiation and increased cell motility of A549 lung epithelial cells. Eur J Cancer 43: 1214–1224. 17379505 10.1016/j.ejca.2007.01.034

[18] Tsuji T, Ibaragi S, Hu GF (2009) Epithelial-mesenchymal transition and cell cooperativity in metastasis. Cancer Research 69: 7135–7139. 10.1158/0008-5472.CAN-09-1618 19738043 PMC2760965

[19] Sato M, Shames DS, Hasegawa Y (2012) Emerging evidence of epithelial-to-mesenchymal transition in lung carcinogenesis. Respirology 17: 1048–1059. 10.1111/j.1440-1843.2012.02173.x 22452538

[20] Krohn A, Ahrens T, Yalcin A, Plones T, Wehrle J, Taromi S, et al (2014) Tumor cell heterogeneity in Small Cell Lung Cancer (SCLC): phenotypical and functional differences associated with Epithelial-Mesenchymal Transition (EMT) and DNA methylation changes. PLoS One 9: e100249 10.1371/journal.pone.0100249 24959847 PMC4069054

[21] Canadas I, Taus A, Gonzalez I, Villanueva X, Gimeno J, Pijuan L, et al (2014) High circulating hepatocyte growth factor levels associate with epithelial to mesenchymal transition and poor outcome in small cell lung cancer patients. Oncotarget 5: 5246–5256. 25026301 10.18632/oncotarget.2124 PMC4170595

[22] Pettengill OS, Sorenson GD, Wurster-Hill DH, Curphey TJ, Noll WW, Cate CC, et al (1980) Isolation and growth characteristics of continuous cell lines from small-cell carcinoma of the lung. Cancer 45: 906–918. 6266631 10.1002/1097-0142(19800301)45:5<906::aid-cncr2820450513>3.0.co;2-h

[23] White A, Stewart MF, Farrell WE, Crosby SR, Lavender PM, Twentyman PR, et al (1989) Pro-opiomelanocortin gene expression and peptide secretion in human small-cell lung cancer cell lines. Journal of Molecular Endocrinology 3: 65–70. 2472813 10.1677/jme.0.0030065

[24] West MJ (2012) Introduction to stereology. Cold Spring Harb Protoc 2012. 10.1101/pdb.top070623 22854572

[25] Yamamoto H, Yamane T, Iguchi K, Tanaka K, Iddamalgoda A, Unno K, et al (2015) Melanin production through novel processing of proopiomelanocortin in the extracellular compartment of the auricular skin of C57BL/6 mice after UV-irradiation. Sci Rep 5: 14579 10.1038/srep14579 26417724 PMC4586518

[26] Burrows N, Telfer B, Brabant G, Williams KJ (2013) Inhibiting the phosphatidylinositide 3-kinase pathway blocks radiation-induced metastasis associated with Rho-GTPase and Hypoxia-inducible factor-1 activity. Radiother Oncol. 10.1016/j.radonc.2013.06.027 23891094

[27] Zhang X, Li X, Zhang N, Yang Q, Moran MS (2011) Low doses ionizing radiation enhances the invasiveness of breast cancer cells by inducing epithelial-mesenchymal transition. Biochem Biophys Res Commun 412: 188–192. 10.1016/j.bbrc.2011.07.074 21810413

[28] Dasari V, Gallup M, Lemjabbar H, Maltseva I, McNamara N (2006) Epithelial-mesenchymal transition in lung cancer: is tobacco the "smoking gun"? Am J Respir Cell Mol Biol 35: 3–9. 16484682 10.1165/rcmb.2006-0051SF

[29] Radisky DC (2005) Epithelial-mesenchymal transition. J Cell Sci 118: 4325–4326. 16179603 10.1242/jcs.02552

[30] Hanahan D, Weinberg RA (2011) Hallmarks of cancer: the next generation. Cell 144: 646–674. 10.1016/j.cell.2011.02.013 21376230

