# Supplementary figures and images for: Irradiation Decreases the Neuroendocrine Biomarker Pro-Opiomelanocortin in Small Cell Lung Cancer Cells In Vitro and In Vivo
Source: PLoS One. 2016 Feb 5;11(2):e0148404. doi: 10.1371/journal.pone.0148404 (PMC4746075; doi:10.1371/journal.pone.0148404)

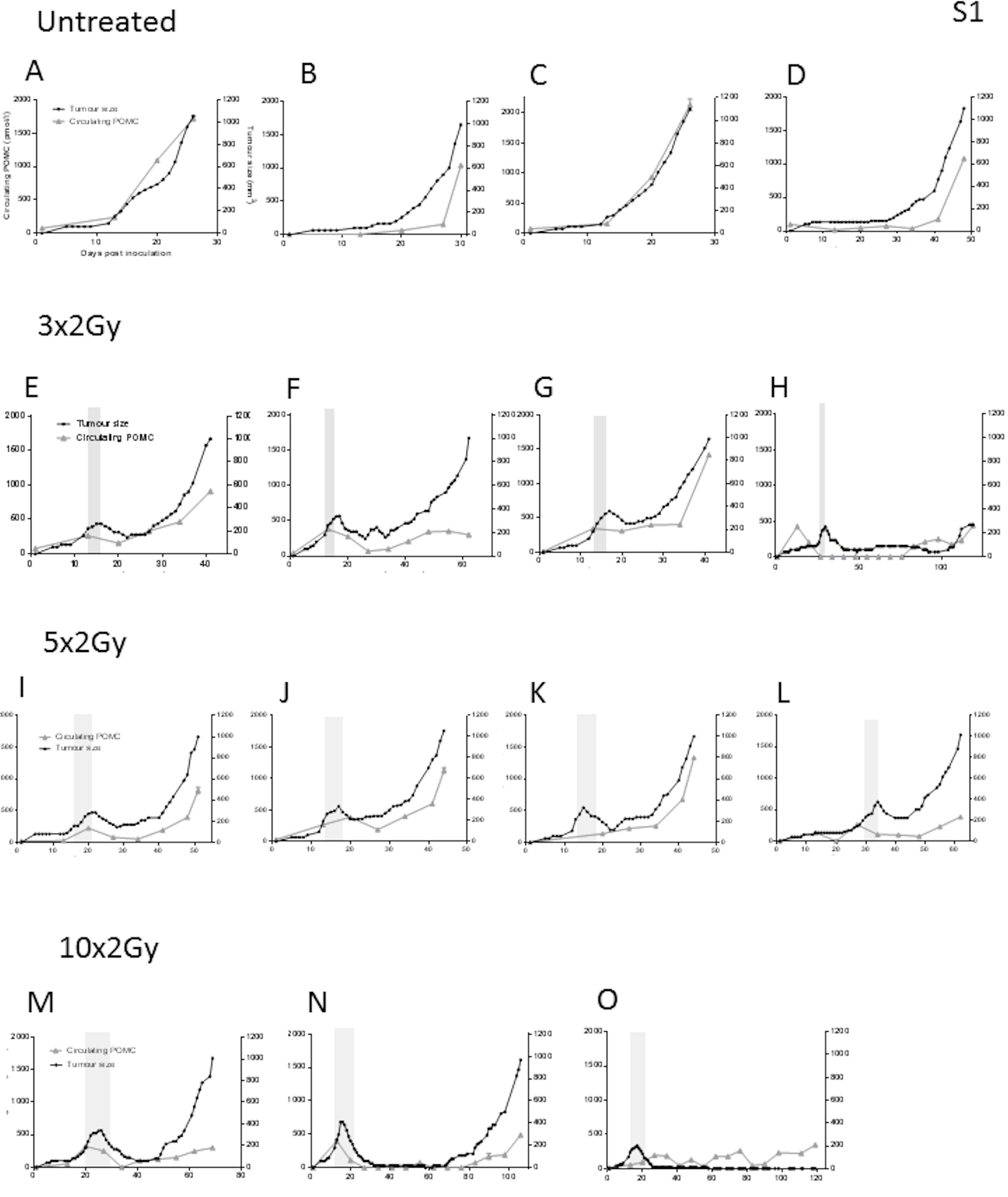

Supplement: S1 Fig — Individual mouse data for DMS 79 xenografts. DMS 79 cells were established as subcutaneous xenografts and once they were 200-250mm3 either left to grow untreated (A-D) or exposed to 2Gy IR/day for 3 consecutive days (E-H), 5 consecutive days (I-L) or 10 consecutive days (M-O). Circulating POMC was monitored by blood sampling on days 0, 13, 20 and every 7 days thereafter. Shaded bars indicate the period where tumours were locally exposed to IR. (TIF) [file pone.0148404.s001.tif]

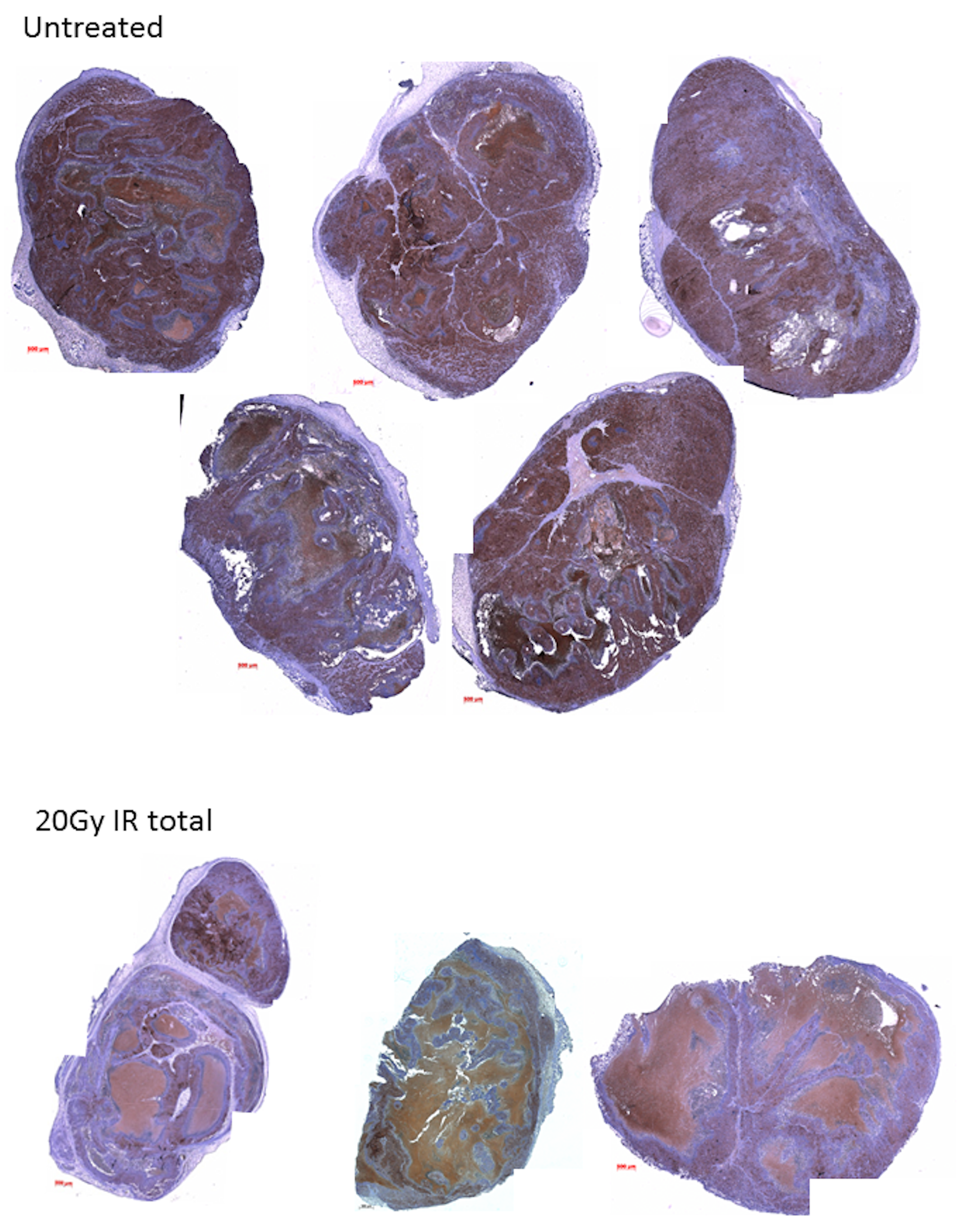

Supplement: S2 Fig — DMS 79 cells were established as subcutaneous tumours in nude mice and either left untreated or exposed to IR for 10 consecutive days at 2Gy/day. A central section of the tumour was stained for POMC using our own N1C11 antibody. In two mice the tumours did not regrow after treatment so POMC could not be assessed (one from group 2 [3 consecutive IR days] and one from group 4 [10 consecutive IR days). Quantitive assessment by positive pixel analysis of untreated tumours and 20Gy IR treated tumours stained for POMC is presented in Fig 2. (TIF) [file pone.0148404.s002.tif]

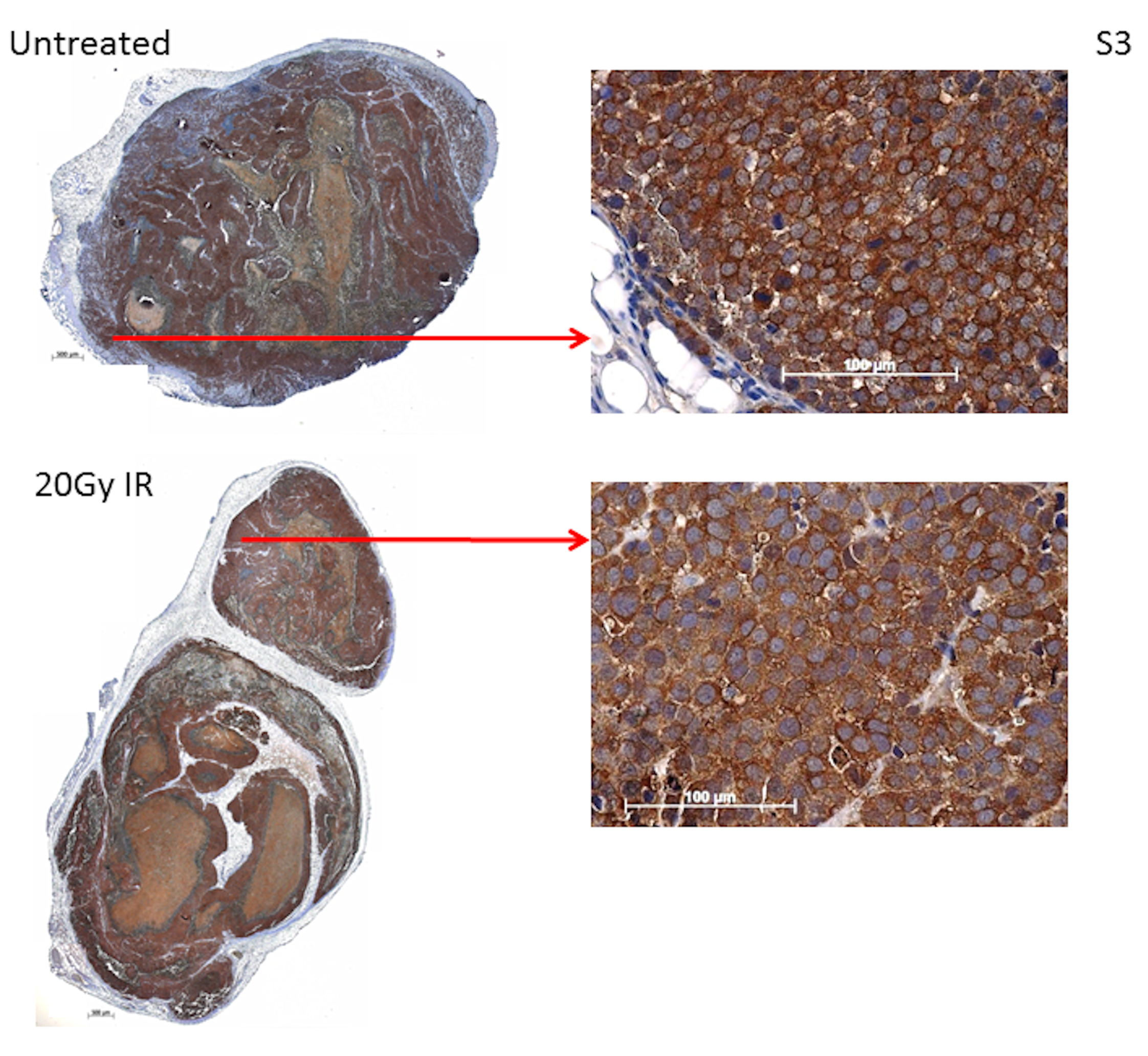

Supplement: S3 Fig — DMS 79 cells were established subcutaneously in nude mice and either left untreated or exposed to IR for 10 days at 2Gy/day. A central section of the tumour was stained for neuron specific enolase (NSE). Tumours presented are representative of 3-5/group. (TIF) [file pone.0148404.s003.tif]
